# Exploring Tumor Heterogeneity: Radiogenomic Assessment of ADFP in Low WHO/ISUP Grade Clear Cell Renal Cell Carcinoma

**DOI:** 10.3390/cancers16183164

**Published:** 2024-09-15

**Authors:** Federico Greco, Andrea Panunzio, Valerio D’Andrea, Mariavittoria Vescovo, Alessandro Tafuri, Simone Carotti, Bruno Beomonte Zobel, Carlo Augusto Mallio

**Affiliations:** 1 Department of Radiology, Cittadella della Salute, Azienda Sanitaria Locale di Lecce, Piazza Filippo Bottazzi, 2, 73100 Lecce, Italy; 2 Research Unit of Radiology, Department of Medicine and Surgery, Università Campus Bio-Medico di Roma, Via Alvaro del Portillo, 21, 00128 Roma, Italy; valerio.dandrea@unicampus.it (V.D.); b.zobel@policlinicocampus.it (B.B.Z.); c.mallio@policlinicocampus.it (C.A.M.); 3 Department of Urology, “Vito Fazzi” Hospital, Piazza Filippo Muratore, 1, 73100 Lecce, Italy; panunzioandrea@virgilio.it (A.P.); tafuri.alessandro@gmail.com (A.T.); 4 Fondazione Policlinico Universitario Campus Bio-Medico, Via Alvaro del Portillo, 200, 00128 Roma, Italy; 5 Anatomical Pathology Operative Research Unit, Fondazione Policlinico Universitario Campus Bio-Medico, Via Alvaro del Portillo, 200, 00128 Roma, Italy; m.vescovo@policlinicocampus.it; 6 Microscopic and Ultrastructural Anatomy Research Unit, Medicine and Surgery, Università Campus Bio-Medico di Roma, Via Alvaro del Portillo, 21, 00128 Roma, Italy; s.carotti@policlinicocampus.it; 7 Predictive Molecular Diagnostics, Fondazione Policlinico Universitario Campus Bio-Medico, Via Alvaro del Portillo, 200, 00128 Roma, Italy

**Keywords:** adipose differentiation-related protein, adipose tissue, clear cell renal cell carcinoma, computed tomography, lipid metabolism, radiogenomics

## Abstract

**Simple Summary:**

This research aims to explore how specific imaging features on computed tomography (CT) scans, related to lipid metabolism, can be linked to a protein called adipose differentiation-related protein (ADFP) in clear cell renal cell carcinoma. This study suggests that tumors with higher lipid content, which can be seen as lower values on a CT scan, are associated with lower-grade, less aggressive cancers that express ADFP. This finding is significant because it could lead to non-invasive methods for evaluating tumor characteristics, potentially helping better understand the nature of the cancer and tailor treatment plans without needing invasive procedures.

**Abstract:**

This study aimed to investigate the association between metabolic lipid computed tomography (CT) features and adipose differentiation-related protein (ADFP) expression in clear cell renal cell carcinoma (ccRCC), providing insights into non-invasive methods for assessing ADFP expression and tumor characteristics. This study utilized data from The Cancer Genome Atlas and the Cancer Imaging Archive to analyze genetic alterations and imaging characteristics in ccRCC patients. Tumoral Hounsfield units (HU) analysis and quantification of abdominal adipose tissue compartments were performed using CT images. Statistical analyses were conducted to compare tumoral HU values according to ADFP gene expression and World Health Organization/International Society of Urological Pathology (WHO/ISUP) tumor grade, as well as to explore correlations between tumoral HU values and adipose tissue quantification. Among the 174 identified patients, those with ADFP gene expression showed significantly lower minimum tumoral HU values in low-grade cancers compared to high-grade cancers. Similarly, patients with low-grade cancers expressing ADFP exhibited lower minimum tumoral HU values compared to those without ADFP expression. Negative correlations were observed between minimum tumoral HU values and visceral adipose tissue, subcutaneous adipose tissue, and total adipose tissue in both ccRCC patients with and without ADFP expression. This study reveals a significant association between metabolic lipid CT features and ADFP expression in ccRCC patients. Lower minimum tumoral HU values, suggestive of higher intracellular lipid accumulation, were observed in tumors with low WHO/ISUP grade and ADFP expression.

## 1. Introduction

Radiogenomics emerges as a novel domain within radiology, correlating imaging characteristics with the genomic composition of diseases [1,2]. The observable imaging phenotypes reflect the larger-scale expression of molecular processes discernible through imaging techniques [1,2]. The inception of radiogenomics owes much to the Human Genome Project, which furnished genomic insights via accessible data resources [3,4].

We now understand that surplus adipose tissue, notably, visceral adipose tissue (VAT), actively contributes to renal cell carcinoma (RCC) pathogenesis. Indeed, elevated VAT levels have been observed in clear cell RCC (ccRCC) [5].

Insufficient oxygen supply to adipocytes prompts the release of hypoxia-inducible factor 1 (HIF-1) from excessive adipose tissue, accompanied by irregular secretion of adipokines such as leptin, adiponectin, resistin, and visfatin. This mechanism could potentially link obesity to the onset of RCC [6,7,8].

Adipose differentiation-related protein (ADFP), crucial for fatty acid uptake and the formation of lipid storage droplets, is highly expressed in ccRCC at both transcriptional and protein levels [9,10,11]. Its elevated expression is also notable in adipocytes [12]. ADFP is a hypoxia-inducible gene, with its transcriptional activation governed by HIF [13]. The von Hippel Lindau protein (VHLp) forms a complex that regulates HIF degradation; mutations in VHL lead to its inactivation, loss of HIF degradation, and, consequently, activation of pro-angiogenic pathways and cell growth [14,15,16,17]. This implies that VHL mutation, by inhibiting HIF inactivation, may contribute to the up-regulation of ADFP in ccRCC [11]. This suggests that the link between VHL mutation and ADFP expression in ccRCC could lead to adopting common therapeutic strategies, such as belzutifan or anthiangiogenic therapies (sunitinib, sorafenib, axitinib, or bevacizumab) [18,19,20].

Yao et al. observed that in low-grade ccRCCs, there is a tendency for higher expression levels of ADFP compared to high-grade tumors. This overexpression of ADFP correlates with increased intracellular lipid storage in low-grade ccRCCs when compared to high-grade tumors [11,21]. Meanwhile, Choi et al. illustrated that the reduced attenuation observed in low-grade tumors on unenhanced computed tomography (CT) images likely reflects a greater lipid content within these tumors [22].

CcRCC tissues exhibit significant histopathological diversity within the tumor epithelia, characterized by variations in nuclear/nucleolar characteristics, which are fundamental to the clinical grading system [23,24]. Heterogeneity is also present in tumor structure, cellular morphology, and alterations in the microenvironment [25]. High-grade tumors carry a heightened risk of post-surgical disease recurrence and might benefit from intensified surveillance. Recent findings suggest that disparities in cytological patterns are associated with more aggressive disease [25,26].

To have an effective estimate of the number of intracellular lipid droplets using a non-invasive method, bypassing the obstacle of tumor heterogeneity typical of ccRCC, in this study, we acquired the data of the minimum and maximum values of the tumoral Hounsfield units (HU) values present within the region of interest (ROI) in order to search for a metabolic lipid radiogenomic sign of ADFP expression in low World Health Organization/International Society of Urological Pathology (WHO/ISUP) grade ccRCCs.

The aim of this study is to examine the link between the metabolic lipid features observed in CT scans and the expression of ADFP in ccRCC, offering insights into non-invasive approaches for evaluating ADFP expression considering the tumor heterogeneity typical of ccRCC.

## 2. Materials and Methods

### 2.1. The Cancer Genome Atlas

The Cancer Genome Atlas, backed by both the National Cancer Institute and the National Human Genome Research Institute, serves as a comprehensive record of genetic alterations across over 20 cancer types, including ccRCC. All participating institutions provided tissue samples, which underwent multiplatform genomic profiling and analysis following approval by institutional review boards. Additionally, the Cancer Imaging Archive, supported by the National Cancer Institute, functions as an anonymized repository for pretreatment medical images in DICOM format. Through a unique identifier, imaging data from The Cancer Imaging Archive and tissue samples from The Cancer Genome Atlas are interconnected and available for public retrieval [27].

### 2.2. Lipid Metabolism Imaging Features

The assessment of imaging characteristics indicative of ccRCC lipid metabolism involved measuring tumoral HU and quantifying abdominal adipose tissue compartments. Tumoral HU analysis was conducted on unenhanced CT images by placing a ROI within the solid portion of the tumor and recording the minimum and maximum HU values within the ROI. The ROI was subsequently investigated with a smaller ROI (equal to 5%), to verify that these values were included in at least 5% of the ROI. These data provide an approximation of the lipid content within the tumor cells. Adipose tissue typically exhibits HU values ranging from −50 to −100; hence, lower HU values corresponding to the solid tumor component suggest a higher accumulation of intracellular lipids.

Abdominal adipose tissue, including TAT, VAT, and subcutaneous adipose tissue (SAT), was quantified using a semi-automatic feature within Horos v.4.0.0 RC2 software. This functionality enabled the analysis of all cross-sectional CT images by identifying the characteristic HU values of adipose tissue. The measurements were acquired as areas (cm^2^) from a single axial image positioned 3 cm above the lower boundary of L3, as detailed previously [28]. All the ROIs were performed by the consensus of two radiologists (F. G. and C. A. M., with 9 years and 13 years of experience, respectively), who were blinded to the clinical data.

### 2.3. Statistical Methods

Three sets of analyses were performed. First, we tabulated the demographics and clinical–pathological characteristics of the entire patient population (*n* = 174). Descriptive statistics included frequencies and proportions for categorical variables; medians and interquartile ranges (IQRs) were reported for continuously coded variables. Second, minimum and maximum values for tumoral HU were compared according to ADFP gene expression (yes vs. no) and WHO/ISUP tumor grade (low-grade [G1–2] vs. high-grade [G3–4]). Finally, correlation analysis was used to test the relationship between minimum tumoral HU values and quantification of VAT, SAT, and TAT according to ADFP expression. All tests were two-sided with a level of significance set at *p* < 0.05. The R software environment for statistical computing and graphics (version 4.1.2, R Foundation for Statistical Computing, Vienna, Austria) was used for all analyses.

## 3. Results

The main characteristics of 174 identified patients are listed in Table 1. Overall, median age was 59 (51, 69) years, 114 (65.5%) patients were males, 107 (61.4%) harbored a localized disease, and 68 (39.1%) presented with a low WHO/ISUP tumor grade (G1–2) at final pathology. Forty (23.0%) subjects had ADFP gene expression. The median for minimum and maximum tumoral HU values was −5 (−18, 3) and 78 (67, 91), respectively.

Among patients without ADFP expression (*n* = 134), no statistically significant differences emerged between low-grade and high-grade cancers for either minimum or maximum tumoral HU values (Table 2). Conversely, among patients with ADFP gene expression (*n* = 40), those harboring low-grade cancers had lower minimum tumoral HU values compared to those harboring high-grade cancers (−23 vs. −3, *p* = 0.002).

Among patients with low-grade cancers (*n* = 68), those who did express ADFP had lower minimum tumoral HU values compared to those who did not express ADFP (−23 vs. −6, *p* = 0.005; Table 2). Conversely, among subjects with high-grade cancers (*n* = 106), no statistically significant differences emerged between patients with and without ADFP expression for either minimum or maximum tumoral HU values.

**Figure 1:**
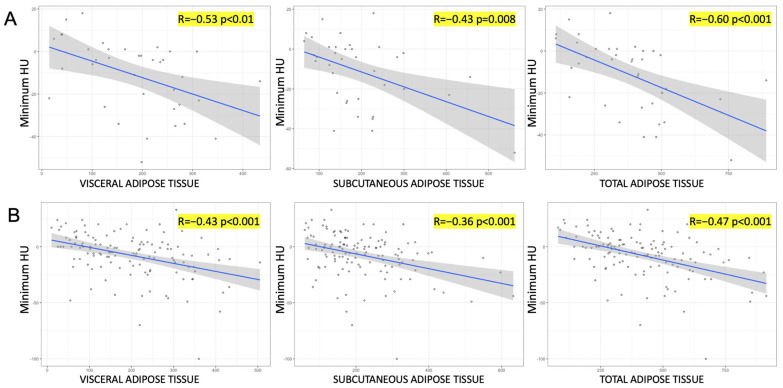
Scatterplots illustrating the relationship between minimum tumoral HU and quantification of adipose tissue compartments according to ADFP expression [(**A**): yes vs. (**B**): no].

Figure 1 shows the relationship between minimum tumoral HU and quantification of adipose tissue compartments according to ADFP expression. Accordingly, a negative correlation was observed between minimum tumoral HU values and VAT, SAT, and TAT in both groups. However, this relationship was stronger in patients who expressed ADFP compared to those who did not (R coefficient: −0.57 vs. −0.43 for VAT; −0.43 vs. −0.36 for SAT; −0.60 vs. −0.47 for TAT).

## 4. Discussion

**Figure 2:**
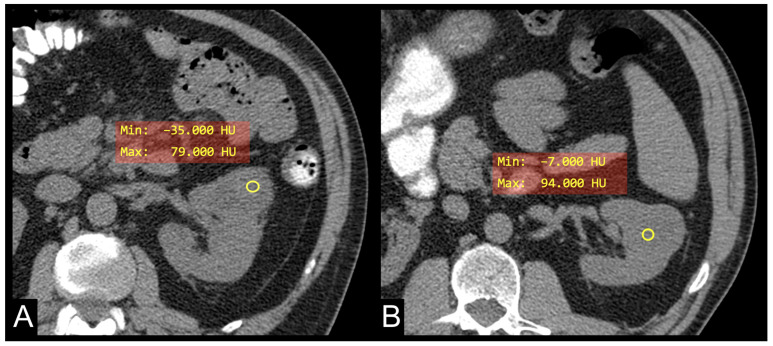
Unenhanced axial CT images of patients with low WHO/ISUP grade ccRCC with ADFP expression (**A**) and low WHO/ISUP grade ccRCC without ADFP expression (**B**) show yellow ROIs with different minimum tumor HU values (HU −35 and −7, respectively).

In this study, we examined the metabolic lipid CT characteristics associated with ADFP expression in ccRCC patients. We observed a notable reduction in minimum tumoral HU values among ccRCC patients with low WHO/ISUP grade compared to those with low WHO/ISUP grade but without ADFP expression (*p* = 0.005) (Figure 2). Additionally, there was a significant decrease in minimum tumoral HU values in ccRCC patients with low WHO/ISUP grade and ADFP expression in comparison to those with high WHO/ISUP grade and ADFP expression (*p* = 0.002). Furthermore, we found a negative correlation between minimum tumor HU values and VAT, SAT, and TAT in ccRCC patients with ADFP expression (*p* < 0.001 for VAT and TAT, *p* = 0.008 for SAT), and without ADFP expression (*p* < 0.001 for VAT, SAT, and TAT), showing a stronger relationship in ccRCC patients with ADFP expression.

**Figure 3:**
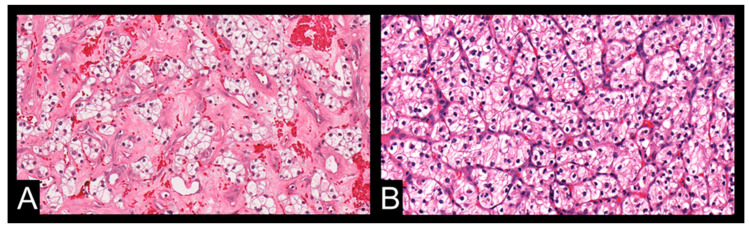
The histological images illustrate two samples of ccRCC (same patients of Figure 2) stained with hematoxylin and eosin (original magnification 20×). Image (**A**): ccRCC with ADFP expression characterized by cells with nested architecture, small nuclei, inconspicuous nucleoli, and abundant clear cytoplasm. Image (**B**): ccRCC without ADFP expression characterized by cells with granular eosinophilic cytoplasm.

The findings from this study suggest a compelling link between metabolic lipid CT features and ADFP expression in ccRCC patients. The observation of a notable reduction in minimum tumoral HU values among ccRCC patients with low WHO/ISUP grade and ADFP expression compared to those without ADFP expression underscores the significance of intracellular lipid accumulation in this context. In the case of ccRCC, lower HU values typically represent an estimate of higher lipid content within the tumor and could reflect the phenotypic counterpart of ADFP activity involved in the uptake of fatty acids and the formation of lipid storage droplets (Figure 3).

In ccRCC, ADFP expression might play a role in the buildup of lipid droplets within tumor cells, a characteristic frequently observed in this histotype [11]. CcRCC is recognized for its heightened lipid content, encompassing cholesterol, cholesterol esters, and phospholipids in the cytoplasm. This accumulation results in a distinctive ‘yellow’ appearance upon macroscopic examination and predominantly comprises ‘clear cells’ when subjected to routine haematoxylin–eosin staining [29,30].

The study of Yao et al. corroborates the prior observation that ADFP exhibited up-regulation at both transcriptional and protein levels within the cancerous cells of ccRCC. Meanwhile, ADFP protein levels were nearly indiscernible via immunohistochemistry in normal kidney tissues, including proximal tubular epithelial cells, which are recognized as precursor cells for the clear cell subtype of RCC [11,18,27]. Additionally, in ccRCCs with a lower nuclear grade (G1 and G2), there is a characteristic ‘clear-cell’ appearance. However, as the nuclear grade rises (G3 and G4), the cytoplasm becomes more eosinophilic, and its ‘clear-cell’ nature diminishes [30]. It has been demonstrated that ADFP is overexpressed at both mRNA and protein levels in ccRCC (18). Immunohistochemically, the ADFP protein was abundant in the cytoplasm and near the cell membrane of tumor cells in the majority of ccRCCs, consistent with its previously observed location in lipid-accumulating adipocytes [31]. Yao et al. also observed that low-grade ccRCCs tend to exhibit higher ADFP mRNA and protein expression compared to high-grade tumors. These findings strongly indicate that the overexpression of ADFP is involved in lipid uptake and storage in ccRCC [21]. Furthermore, ADFP expression levels are likely indicative of the macroscopic and microscopic morphological features of ccRCC [21]. Consequently, these levels were markedly lower in tumors exhibiting a spindle/pleomorphic component, indicative of dedifferentiated histology associated with heightened aggressiveness and potential for metastasis [21,30,32,33,34]. The prevailing belief is that cancer transformation, tumor progression, and the development of aggressive and metastatic capabilities involve the accumulation of multiple genetic alterations over time [35,36]. The findings of Yao et al. indicate that, following its transcriptional activation in an early stage, ADFP is subsequently down-regulated as part of the later dedifferentiation processes during the tumorigenesis of ccRCC [21].

While the reduction in minimum tumoral HU values among ccRCC patients with low WHO/ISUP grade and ADFP expression suggests a potential non-invasive approach to assessing ADFP expression, it is essential to acknowledge that determining WHO/ISUP grade typically requires invasive methods such as histopathological examination of tissue samples obtained through biopsy or surgical resection. Radiological features play a crucial role in detecting tumor grade in ccRCC. One such feature is the presence of peritumoral collateral vessels, which have been linked to high tumor grade in ccRCC. This association suggests that the absence of these vessels could potentially indicate a lower tumor grade [37,38]. From this, it can be deduced that ccRCC patients without peritumoral collateral vessels may be candidates for radiogenomic analysis to evaluate ADFP expression.

Overall, ccRCC patients without peritumoral collateral vessels may represent a subgroup that could benefit from radiogenomic analysis to evaluate ADFP expression, offering a potential non-invasive approach for assessing tumor characteristics and guiding personalized treatment strategies.

Yao et al. observed higher ADFP expression levels in low-grade ccRCCs compared to high-grade tumors, correlating this overexpression with increased intracellular lipid storage [11,21]. Meanwhile, Choi et al. noted reduced attenuation on unenhanced CT images of low-grade tumors, indicative of greater lipid content within these tumors [22]. These observations align with the current study’s findings, which reveal a notable reduction in minimum tumoral HU values among ccRCC patients with low WHO/ISUP grade and ADFP expression, suggesting a higher accumulation of intracellular lipids in these tumors.

The negative correlation between minimum tumor HU values and abdominal adipose tissue compartments in ccRCC patients, both with and without ADFP expression, suggests that tumors with lower HU values, indicative of higher lipid content, are linked to increased fat deposits in the body. The correlation is stronger in patients with ADFP expression, in line with the activity of ADFP linked to lipid metabolism within tumors. This highlights ADFP’s role in lipid accumulation, which is particularly significant in lower-grade ccRCC, where intracellular lipid storage is more prominent.

We acknowledge several limitations of this study that could affect the interpretation and generalizability of its findings. Firstly, the small number of patients included in this study may limit its statistical power and the reliability of the observed associations. Moreover, confounding factors related to the expression of other genes associated with the accumulation of lipid droplets, such as Ancient Ubiquitous Protein 1 (AUP1) and Acyl-CoA Synthetase 3 (ASCL3), were not fully accounted for [39,40]. Future studies with larger sample sizes and without the presence of confounding gene expressions are warranted to further elucidate the role of ADFP in ccRCC. Additionally, the retrospective design of this study could introduce selection bias and limit the ability to establish causal relationships between ADFP expression and imaging features. Furthermore, the variability in imaging protocols and equipment among different institutions was not addressed, potentially affecting the reliability and reproducibility of the results. Standardization of imaging techniques across institutions could mitigate this limitation. Biological variability among patients, including variations in tumor biology, metabolism, and risk factors, could confound the observed associations between ADFP expression, imaging features, and clinical outcomes. This study did not provide information on certain variables that could influence the relationship between ADFP expression and imaging features, such as patient comorbidities, medications, or lifestyle factors, which could limit the comprehensiveness of the analysis. Thus, cautious interpretation of the results is warranted, and further research addressing these limitations is necessary to strengthen the evidence base in this area.

## 5. Conclusions

This study concludes that there is a significant association between metabolic lipid CT features and ADFP expression in ccRCC patients. Specifically, ccRCC tumors with low WHO/ISUP grade and ADFP expression exhibit a notable reduction in minimum tumoral HU values, suggesting a higher accumulation of intracellular lipids. This finding underscores the potential of non-invasive imaging techniques to assess ADFP expression and tumor characteristics in certain ccRCC patients.

## Figures and Tables

**Table 1:** Descriptive characteristics of the study population.

	Overall *n* = 174 ^1^
**Age (**years)	59 (51, 69)
**Sex** (Males)	114 (65.5%)
**Race/ethnicity** (Caucasian)	164 (94.3%)
**History of cancer**	30 (17.2%)
**Primary tumor size on the axial plane** (mm)	53.0 (37.3, 78.0)
**Laterality** (left)	76 (43.7%)
**Tumor grade (**WHO/ISUP) Low-grade (G1–2) High-grade (G3–4)	68 (39.1%) 106 (60.9%)
**Tumor stage** Stage I Stage II Stage III Stage IV	93 (53.4%) 14 (8.0%) 45 (25.9%) 22 (12.7%)
**ADFP expression**	40 (23.0%)
**Abdominal adipose tissue compartments ** VAT SAT TAT	197.5 (103.2, 270.6) 180.3 (136.9, 272.1) 397.0 (278.9, 505.2)
**HU** Median Minimum Maximum	36 (30, 40) −5 (−18, 3) 78 (67, 91)

^1^ Median (IQR); n (%); Abbreviations: ADFP, Adipose Differentiation-Related Protein; VAT, Visceral Adipose Tissue; SAT, Subcutaneous Adipose Tissue; TAT, Total Adipose Tissue; HU, Hounsfield Units.

**Table 2:** Minimum and maximum tumoral HU values according to ADFP gene expression (yes vs. no) and WHO/ISUP tumor grade (low vs. high).

**No ADFP expression (*n* = 134)**			
	Low-grade *n* = 57 (42.5%) ^1^	High-grade *n* = 77 (57.5%) ^1^	*p*-value ^2^
**HU minimum**	−6 (−16, 4)	−5 (−18, 6)	0.6
**HU maximum**	75 (62, 88)	80 (70, 91)	0.065
**ADFP expression (*n* = 40)**			
	Low-grade *n* = 11 (27.5%) ^1^	High-grade *n* = 29 (72.5%) ^1^	*p*-value ^2^
**HU minimum**	−23 (−38, −13)	−3 (−11, 1)	0.002
**HU maximum**	71 (63, 100)	79 (69, 91)	0.9
**Low-grade (*n* = 68)**			
	ADFP expression *n* = 11 (16.2%) ^1^	No ADFP expression *n* = 57 (83.8%) ^1^	*p*-value ^2^
**HU minimum**	−23 (−38, −13)	−6 (−16, 4)	0.005
**HU maximum**	71 (63, 100)	75 (62, 88)	0.5
**High-grade (*n* = 106)**			
	ADFP expression *n* = 29 (27.4%) ^1^	No ADFP expression *n* = 77 (72.6%) ^1^	*p*-value ^2^
**HU minimum**	−3 (−11, 1)	−5 (−18, 6)	0.9
**HU maximum**	79 (69, 91)	80 (70, 91)	0.6

^1^ Median (IQR); n (%) ^2^ Wilcoxon rank sum exact test. Abbreviations: ADFP, Adipose Differentiation-Related Protein; HU, Hounsfield Units. Values in bold indicate statistical significance set at *p* < 0.05.

## Data Availability

The data presented in this study are openly available in The Cancer Imaging Archive (https://www.cancerimagingarchive.net/collection/tcga-kirc/, accessed on 1 November 2019).
